# Protective effects of *APOE e2* against disease progression in subcortical vascular mild cognitive impairment patients: A three-year longitudinal study

**DOI:** 10.1038/s41598-017-02046-y

**Published:** 2017-05-15

**Authors:** Yeo Jin Kim, Sang Won Seo, Seong Beom Park, Jin Ju Yang, Jin San Lee, Juyoun Lee, Young Kyoung Jang, Sung Tae Kim, Kyung-Han Lee, Jong Min Lee, Jae-Hong Lee, Jae Seung Kim, Duk L. Na, Hee Jin Kim

**Affiliations:** 1 Department of Neurology, Chuncheon Sacred Heart Hospital, Hallym University College of Medicine, Chuncheon, Korea; 2 Department of Neurology, Sungkyunkwan University School of Medicine, Samsung Medical Center, Seoul, Korea; 3 Neuroscience Center, Samsung Medical Center, Seoul, Korea; 4 Department of Biomedical Engineering, Hanyang University, Seoul, Korea; 5 Department of Neurology, Kyung Hee University Hospital, Seoul, Korea; 6 Department of Neurology, Chungnam National University Hospital, Daejeon, Korea; 7 Department of Radiology, Sungkyunkwan University School of Medicine, Samsung Medical Center, Seoul, Korea; 8 Department of Nuclear Medicine, Sungkyunkwan University School of Medicine, Samsung Medical Center, Seoul, Korea; 9 Department of Neurology, Asan Medical Center, University of Ulsan College of Medicine, Seoul, Korea; 10 Department of Nuclear Medicine, Asan Medical Center, University of Ulsan College of Medicine, Seoul, Korea; 11 Department of Health Sciences and Technology, SAIHST, Sungkyunkwan University, Seoul, Korea; 12 Department of Clinical Research Design & Evaluation, SAIHST, Sungkyunkwan University, Seoul, Korea

## Abstract

Although the association between apolipoprotein E (APOE) genotype and disease progression is well characterized in patients with Alzheimer’s disease, such a relationship is unknown in patients with subcortical vascular cognitive impairment. We evaluated whether APOE genotype is associated with disease progression in subcortical vascular mild cognitive impairment (svMCI) patients. We prospectively recruited 72 svMCI patients (19 *APOE4* carriers, 42 *APOE3 homozygotes*, and 11 *APOE2* carriers). Patients were annually followed-up with brain MRI and neuropsychological tests for three years and underwent a second Pittsburgh compound B (PiB)-PET at a mean interval of 32.3 months. Amyloid-ß burden was quantified by PiB standardized uptake value ratio (SUVR), and the amount of small vessel disease was quantified by number of lacune and small vessel disease score on MRI. We also measured cortical thickness. During the three years of follow-up, compared to the *APOE3 homozygotes*, there was less increase in PiB SUVR among *APOE2* carriers (p = 0.023), while the APOE genotype did not show significant effects on small vessel disease progression. *APOE2* carriers also showed less cortical thinning (p = 0.023) and a slower rate of cognitive decline (p = 0.009) compared to those with *APOE3 homozygotes*. Our findings suggest that, in svMCI patients, *APOE2* has protective effects against amyloid-ß accumulation, cortical thinning, and cognitive decline.

## Introduction

Although the association between apolipoprotein E (APOE) genotype and disease progression is well characterized in Alzheimer’s disease (AD) patients, such a relationship is largely unknown in patients with subcortical vascular cognitive impairment, which indicates cognitive impairment related to extensive small vessel disease (SVD). In AD, it is well known that the APOE e4 allele (APOE4) is associated with increased amyloid-ß, rapid cortical thinning^1^, and accelerated cognitive decline^2^, while the APOE e2 allele (APOE2) is associated with less amyloid-ß^3^, slower cortical thinning^4^, and slower cognitive decline^3^. However, those relationships have not been studied in patients with extensive SVD.

We evaluated whether APOE genotype affected the progression of amyloid-ß, SVD burden, cortical thinning, and cognitive decline in a longitudinal cohort of patients with subcortical vascular mild cognitive impairment (svMCI). In our previous cross-sectional study of subcortical vascular cognitive impairment patients, APOE4 was associated with higher amyloid-ß burden, while it was not associated with SVD markers such as lacune or white matter hyperintensities (WMH)^5^. In a longitudinal study of subcortical vascular dementia patients, co-associated amyloid-ß was reported to have strong effects on cortical thinning and cognitive decline^6^. Therefore, we hypothesized that APOE2 would decelerate and APOE4 would accelerate amyloid-ß accumulation, whereas the rate of SVD progression would not be affected by APOE genotype. In addition we further hypothesized that the rate of cortical thinning and cognitive decline would be slower in APOE2 and faster in APOE4 carriers.

## Results

### Baseline demographic and clinical characteristics

Compared to APOE3 homozygotes, APOE4 carriers had smaller WMH volume at baseline. The number of lacune and cortical thickness were not different among the three groups at baseline (Table 1).

**Table 1:** Baseline demographic and clinical characteristics.

	APOE3 homozygote (n = 42)	APOE2 carrier (n = 11)	APOE4 carrier (n = 19)	p-value^*^	p-value^†^	p-value^‡^
Age^a^	74.5 ± 7.3	74.1 ± 3.9	73.0 ± 7.4	0.999	0.999	0.999
Gender^b^ (%, female)	26 (61.9)	7 (63.6)	12 (63.2)	0.999	0.999	0.999
Education^a^	8.9 ± 4.5	6.8 ± 6.6	9.5 ± 5.7	0.738	0.999	0.540
Vascular risk factor^b^						
Hypertension	34 (81.0)	8 (72.7)	12 (63.2)	0.999	0.405	0.999
Diabetes	11 (26.2)	1 (9.1)	6 (31.6)	0.683	0.999	0.482
Hyperlipidemia	17 (40.5)	4 (36.4)	4 (21.1)	0.999	0.418	0.999
Image markers^a^						
PiB SUVR	1.38 ± 0.30	1.61 ± 0.51	1.61 ± 0.41	0.197	0.085	0.999
Lacune﻿﻿, No﻿.	4.5 (0–28)	2 (0–24)	3 (0–19)	0.999	0.758	0.999
SVD score	4.0 (1–4)	3.5 (1–4)	3 (1–4)	0.999	0.526	0.999
WMH volume, mL	32.2 ± 17.6	39.4 ± 19.3	17.7 ± 40.4	0.999	0.040	0.231
Cortical thickness, mm	2.83 ± 0.13	2.79 ± 0.15	2.86 ± 0.18	0.999	0.999	0.823
K-MMSE^a^	26.6 ± 2.6	25.3 ± 4.0	26.1 ± 2.6	0.531	0.999	0.999
CDR-SOB^a^	1.30 ± 0.78	1.14 ± 0.78	1.29 ± 0.82	0.999	0.999	0.999

ANOVA test for continuous variables and Chi square test for categorical variables were conducted. To correct for multiple comparisons, we performed Bonferroni post hoc analysis.

^*^Comparison of APOE3 homozygotes vs. APOE2 carriers.

^†^Comparison of APOE3 homozygotes vs. APOE4 carriers.

^‡^Comparison of APOE2 vs. APOE4 carriers.

^a^Continuous variables are expressed as mean ± standard deviation or median (minimum-maximum) if not normally distributed.

^b^Categorical variables are expressed as prevalence (%).

APOE, apolipoprotein E; PiB = Pittsburgh compound B; SUVR = standardized uptake value ratio; No. = number; SVD = small vessel disease; WMH = white matter hyperintensity; K-MMSE = Korean version of the Mini-Mental State Examination; CDR-SOB = Clinical Dementia Rating-Sum of Boxes.

### Relationships between APOE genotype and longitudinal changes in PiB SUVR, lacune number, and SVD score over 3 years

Compared to APOE3 homozygotes, APOE2 carriers showed a slower increase in PiB SUVR (p = 0.023), especially in the frontal, parietal, and temporal regions (Table 2). This relationship remained to be significant after further adjusting for baseline WMH volume (ß (SE) = −0.06 (0.026), p = 0.021). There were no effects of APOE genotype on the progression of lacune number or SVD score (Table 2).

**Table 2:** Relationships between APOE genotype and the progression rate of PiB SUVR, lacune number, or SVD score.

	APOE3 homozygote	APOE2 carrier		APOE4 carrier	
		ß (SE)	p value	ß (SE)	p value
Amyloid burden (PiB SUVR)					
Global	Ref	−0.06 (0.027)	0.023	0.028 (0.026)	0.27
Frontal	Ref	−0.06 (0.027)	0.037	0.029 (0.029)	0.316
Parietal	Ref	−0.06 (0.028)	0.041	0.04 (0.028)	0.124
Temporal	Ref	−0.07 (0.024)	0.004	0.02 (0.023)	0.388
Cingulate	Ref	−0.06 (0.031)	0.06	0.03 (0.026)	0.339
SVD burden					
Lacune, No	Ref	−0.02 (0.046)	0.638	0.04 (0.050)	0.394
SVD score	Ref	0.03 (0.025)	0.204	0.01 (0.020)	0.55

Generalized estimating equation with linear model was conducted with regional PiB SUVR, lacune number, or SVD score as the dependent variables and APOE genotype as the independent variables after controlling for age and gender.

APOE = apolipoprotein E; PiB = Pittsburgh compound B; SUVR = standardized uptake value ratio; SVD = small vessel disease; No. = number; β = unstandardized beta coefficient; SE = standard error; Ref = reference.

### Relationships between APOE genotype and the rate of cortical thinning and cognitive decline over 3 years

**Figure 1:**
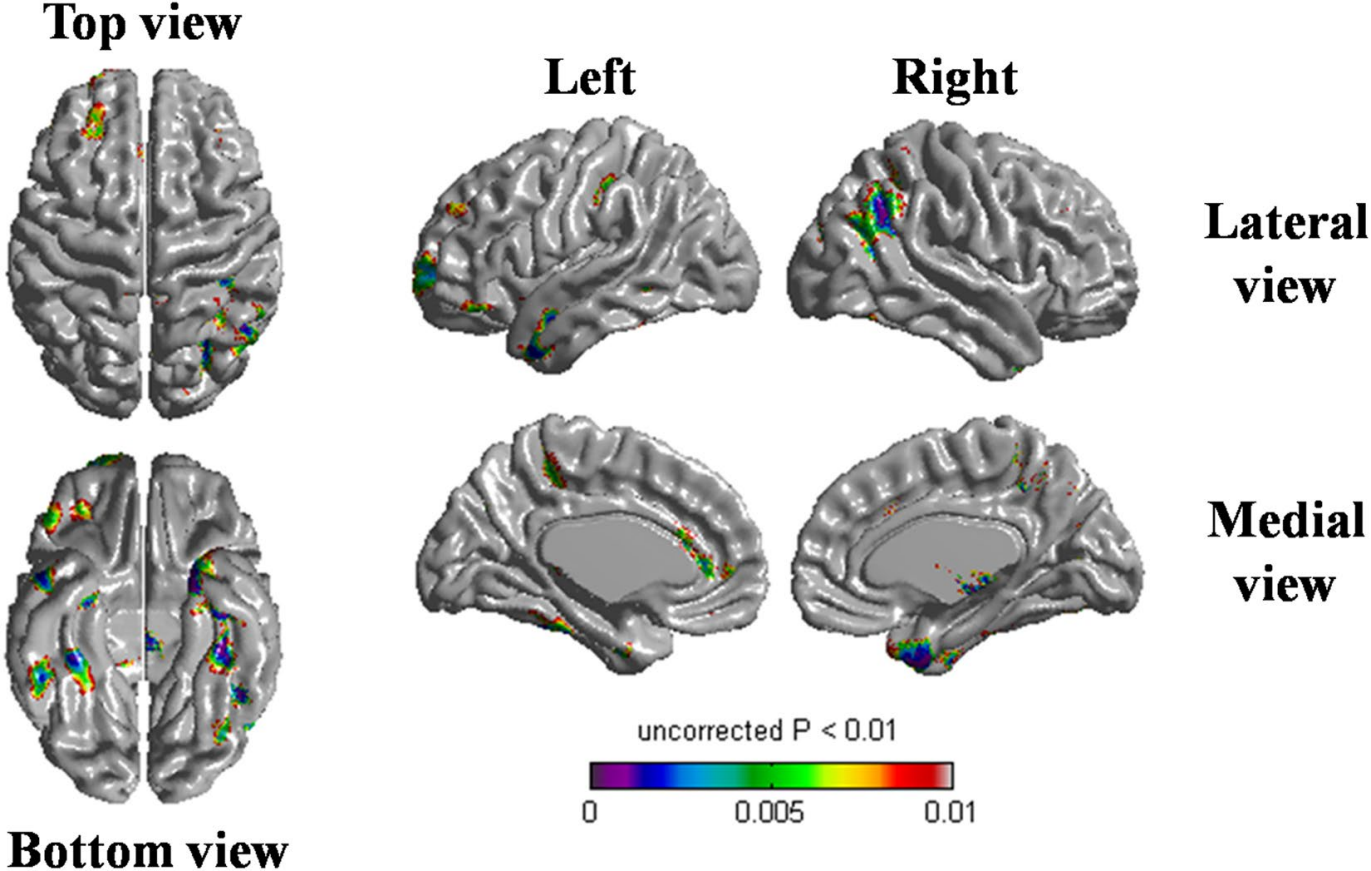
Statistical map shows regions where APOE2 carriers had slower rate of cortical thinning compared to APOE3 homozygotes. Compared to the APOE3 homozygotes, APOE2 carriers showed slower cortical thinning in the left dorsolateral frontal, lateral temporal, medial frontal; right lateral parietal, medial temporal; and bilateral inferior temporal areas. Linear mixed effects model was performed using group (APOE genotype), time, age, gender, intracranial volume, and the interaction term between group and time (group-by-time) as fixed effects; and patient as a random effect (uncorrected p < 0.01). Abbreviation: APOE, apolipoprotein E.

Compared to the APOE3 homozygotes, APOE2 carriers showed slower cortical thinning (p = 0.023), especially in the left dorsolateral frontal, lateral temporal, medial frontal; right lateral parietal, medial temporal; and bilateral inferior temporal areas after adjusting for age, gender and ICV (Fig. 1). This relationship remained to be significant after further adjusting for baseline WMH volume (Supplementary Figure S1).

Compared to APOE3 homozygotes, APOE2 carriers showed a slower cognitive decline as measured by K-MMSE (p = 0.009). Detailed neuropsychological testing revealed a slower decline in language function in APOE2 carriers than in APOE3 homozygotes (Table 3). These relationships remained to be significant after further adjusting for baseline WMH volume (ß (SE) = 0.67 (0.279), p = 0.017 for K-MMSE; ß (SE) = 2.0 (0.669), p = 0.003 for language).

**Table 3:** Relationships between APOE genotype and longitudinal changes in cognitive function

	APOE3 homozygote	APOE2 carrier		APOE4 carrier	
		ß (SE)	p value	ß (SE)	p value
K-MMSE	Ref	0.79 (0.299)	0.009	−0.38 (0.516)	0.459
CDR-SOB	Ref	0.15 (0.271)	0.572	0.01 (0.238)	0.973
Language function	Ref	2.01 (0.646)	0.002	0.03 (0.977)	0.976
Visuospatial function	Ref	1.82 (1.103)	0.098	1.09 (0.707)	0.122
Memory function	Ref	0.97 (1.930)	0.616	3.45 (2.015)	0.087
Frontal-executive function	Ref	0.36 (0.990)	0.715	1.71 (1.079)	0.112

Generalized estimating equation with linear model conducted with cognitive function as the dependent variables and APOE genotype as the independent variables after controlling for age, gender, and education.

APOE = apolipoprotein E; β = unstandardized beta coefficient; SE = standard error; Ref = reference; K-MMSE = Korean version of mini-mental state examination; CDR-SOB = clinical dementia rating-sum of box.

## Discussion

In this longitudinal prospective study, we evaluated the relationships between APOE genotype and disease progression in patients with svMCI. We found that APOE2 carriers showed slower amyloid-ß accumulation compared to APOE3 homozygotes, while APOE genotype did not show a significant effect on SVD progression. Furthermore, the rates of cortical thinning and cognitive decline were slower in APOE2 carriers compared to APOE3 homozygotes. Together, these findings suggest that the APOE2 has a protective effect against disease progression in svMCI patients.

We found a protective effect of the APOE2 on amyloid-ß accumulation in svMCI patients. This is consistent with previous studies with cognitively normal elderlies or AD patients which showed that APOE2 is associated with less amyloid-ß accumulation^7, 8^. Our finding suggests that the protective mechanism of APOE2 remained significant in the presence of extensive SVD. Therefore, the previously reported protective mechanism of APOE2 in AD models might also be applied in svMCI. Previous animal studies showed several mechanisms where APOE2 caused less amyloid-ß formation and enhanced amyloid-ß degradation^9, 10^. Human APOE2 isoform expressed in APP transgenic mice showed that it had the ability to prevent the conversion of amyloid-ß into oligomeric forms^9^. Another animal study showed that APOE2 mice were more effective in reducing amyloid-ß from their bloodstream than APOE4 mice^11^. Previous reports also showed that APOE2 enhanced the degradation of amyloid-ß and formed a more stable complex with amyloid-ß^10, 12^. We suggest that the mechanism described above may also be applied in the setting of severe SVD. Although our hypothesis needs to be confirmed by *in-vitro* or animal studies in the future, we believe that our clinical data provides insight into the protective mechanism of APOE2.

Contrary to our expectation, the rate of amyloid-ß accumulation was not different between APOE4 carriers and APOE3 homozygotes. Although it is well known that the APOE4 impairs amyloid-ß clearance^13^, it might work differently in svMCI patients who have extensive SVD. Animal studies suggested that the presence of extensive SVD may change cerebral blood flow and stiffness of cerebral vessels, which in turn may inhibit amyloid-ß clearance^14, 15^. Further studies are necessary to investigate whether pathomechanism regarding effects of APOE4 on amyloid is modulated by SVD. It is also possible that we did not find a significant relationship due to small sample size. More studies are necessary to elucidate the effects of APOE4 in different neurodegenerative or vascular diseases.

In our cohort of svMCI patients, the APOE genotype was not associated with SVD progression, which is in line with our previous cross-sectional study^5^. Several studies have reported that APOE4 increases risk of having large artery disease^16^ or vascular risk factors such as dyslipidemia, atherogenesis, and coronary atherosclerosis^17–19^. Recent studies have suggested that the APOE4 might contribute to increased risk of vascular dementia^20, 21^. However, the association between APOE genotype and SVD has been controversial. Several studies showed that APOE genotype was associated with SVD markers such as WMH, brain infarcts and cerebral microbleeds^22, 23^, while some studies suggested no association^5, 24^. Our longitudinal data add evidence that APOE genotype is not associated with SVD.

svMCI patients with APOE2 displayed slower cortical thinning and slower cognitive decline compared to APOE3 homozygotes. This is in line with previous studies on healthy elderly or AD patients, in which APOE2 carriers were shown to have less cortical thinning, slower cognitive decline^4, 25^, and have delayed symptom onset^26^. There are several possible mechanisms that account for protective effects of APOE2. Since svMCI patients with APOE2 showed less amyloid-ß accumulation, slower cortical thinning and cognitive decline in svMCI patients with APOE2 might have been mediated by amyloid-ß, which need to be confirmed in further studies with larger sample size. This is supported by a previous study showing that APOE2 had an impact on cognition through its effect on neuritic plaque^3^. On the other hand, APOE2 might have slowed down cortical thinning and cognitive decline through other protective mechanisms such as promoting synaptic integrity, anti-oxidant and anti-inflammatory activities^27–29^. Indeed there are several reports indicating that APOE2 effects on cognitive decline can be independent of amyloid accumulation^30–32^.

This study has several limitations. Firstly, because the sample size was relatively small, the effects of APOE4 might have been missed or the protective effects of APOE2 might be an artifact. However, the protective effects of APOE2 were consistently found in several aspects (amyloid accumulation, cortical thinning, and cognitive decline), which we considered to be meaningful. Secondly, PiB PET can only detect the fibrillary form of amyloid-ß, not soluble amyloid-ß. Lastly, the volume of WMH was not repeatedly measured. However, we used SVD score, which combines the degree of lacune, microbleed, perivascular space, and WMH. Nevertheless, our study is noteworthy in that this is the first longitudinal study that showed the effect of APOE genotype on disease progression in patients with subcortical vascular cognitive impairment. We found that in svMCI patients, APOE2 has a protective effect against amyloid-ß accumulation, cortical thinning, and cognitive decline. Although we did not reveal definite mechanisms through this study, we suggest that our findings improved the understanding of the APOE2 mechanism in the setting of severe SVD, which needs to be confirmed through animal models and clinical studies with larger sample size in the future.

## Methods

### Subjects

We prospectively recruited 72 svMCI patients from September 2008 to September 2011 at Samsung Medical Center. svMCI was diagnosed when patients met the following criteria: 1) a subjective cognitive complaint by the patient or his/her caregiver; 2) normal Activity of Daily Living (ADL) score determined clinically and by the instrumental ADL scale^33^; 3) an objective cognitive decline lower than the 16th percentile on the Seoul Neuropsychological Screening Battery (SNSB); 4) no dementia; 5) a subcortical vascular feature defined as a focal neurological symptom/sign^34^ and 6) significant ischemia on MRI. Significant ischemia was defined as (1) periventricular WMH ≥ 10  mm and (2) deep WMH ≥ 25 mm. svMCI patients were divided into three groups according to APOE genotype: APOE4 carrier (n = 19), APOE2 carrier (n = 11), or APOE3 homozygote (n = 42). There was no subject with APOE e2/e4 genotype.

We obtained written informed consent from each patient. This study was approved by the Institutional Review Board of Samsung Medical Center and the methods were carried out in accordance with the approved guidelines.

### Follow-up evaluations

All patients underwent a clinical interview, a neurological examination, neuropsychological tests, brain MRI, and Pittsburgh compound B (PiB) PET imaging at baseline. Patients were annually followed-up with neuropsychological testing and brain MRI. Of the 72 svMCI patients, 65 completed the first year of follow-up, 51 completed the second year, and 52 (72.2%) completed the third year. In total, 54 (75%) patients underwent a second PiB-PET at a mean interval of 32.3 months. (Supplementary Figure S2).

### Imaging parameters for MRI acquisition

We acquired 3D T1 turbo field echo MR images with the following imaging parameters: sagittal slice thickness, 1.0 mm, over contiguous slices with 50% overlap; no gap; repetition time (TR) of 9.9 msec; echo time (TE) of 4.6 msec; flip angle of 8°; and matrix size of 240 × 240 pixels, reconstructed to 480 × 480 over a field of view (FOV) of 240 mm. The following parameters were used for the 3D FLAIR images: axial slice thickness of 2 mm; no gap; TR of 11,000 msec; TE of 125 msec; flip angle of 90°; and matrix size of 512 × 512 pixels. In whole-brain DT-MRI examinations, sets of axial diffusion-weighted single-shot echo-planar images were collected with the following parameters: 128 × 128 acquisition matrix; 1.72 × 1.72 × 2 mm^3^ voxel size; 70 axial slices; 22 × 22 cm^2^ field of view; TE 60 msec, TR 7696 ms; flip angle 90°; slice gap 0 mm; b-factor of 600 smm^−2^. Diffusion-weighted images were acquired in 45 different directions using the baseline image without weighting [0,0,0]. All axial sections were acquired parallel to the anterior commissure-posterior commissure.

### [11 C] PiB-PET acquisition

[11 C] PiB-PET scanning was performed in a three-dimensional scanning mode that examined 35 slices of 4.25-mm thickness spanning the entire brain. [11 C] PiB was injected into an antecubital vein as a bolus at a mean dose of 420 MBq (i.e., range 259–550 MBq). Sixty minutes after injection, a CT scan was performed for attenuation correction. A 30-minute emission static PET scan was then initiated. The specific radioactivity of [11 C] PiB at the time of administration was greater than 1,500 Ci/mmol for patients, and the radiochemical yield was greater than 35%. The radiochemical purity of the tracer was greater than 95% in all PET studies.

### [11 C] PiB-PET data analysis

PiB PET images were co-registered to individual MRIs, which were normalized to a T1-weighted MRI template. Using these parameters, MRI-co-registered PiB PET images were normalized to the MRI template. The quantitative regional values of PiB retention on the spatially normalized PiB images were obtained through an automated volume of interest (VOI) analysis using the automated anatomical labeling (AAL) atlas. Data processing was performed using SPM Version 5 (SPM5) within Matlab 6.5 (MathWorks, Natick, MA, USA).

We selected 28 cortical VOIs from the left and right hemispheres using the AAL atlas. The cerebral cortical VOIs that were chosen for this study consisted of the bilateral frontal areas (superior and middle frontal gyri; medial portion of the superior frontal gyrus; opercular portion of the inferior frontal gyrus; triangular portion of the inferior frontal gyrus; supplementary motor area; orbital portion of the superior, middle, and inferior orbital frontal gyri; rectus; and olfactory cortex), posterior cingulate gyri, parietal areas (superior and inferior parietal, supramarginal and angular gyri, and precuneus), lateral temporal areas (superior, middle, and inferior temporal gyri; and heschl gyri), and occipital areas (superior, middle, and inferior occipital gyri; cuneus; calcarine fissure; and lingual and fusiform gyri). Regional cerebral cortical uptake ratios were calculated by dividing each cortical VOI uptake ratio by the mean uptake of the cerebellar cortex (cerebellum crus1 and crus2). The global PiB uptake ratio was calculated from the volume-weighted average uptake ratio of 28 bilateral cerebral cortical VOIs. The PiB retention ratio was defined as a continuous variable representing amyloid burden.

### Assessment of amyloid-ß burden

Amyloid-ß burden was assessed using PiB-PET. PiB standardized uptake value ratio (SUVR) was calculated using cerebellar cortex as a reference region.

### Assessment of lacune and total SVD score on MRI

A lacune was defined as a lesion ≥ 3 mm and ≤ 15 mm in diameter with low signal on T1 imaging, high signal on T2-weighted imaging, and a perilesional halo on FLAIR imaging. We rated the total MRI burden of SVD on a scale from 0 to 4, by counting the presence of each of the 4 MRI features of SVD: lacune, microbleed, perivascular space, and WMH^35^.

### Cortical thickness measurement image processing

Cortical thickness was measured using the Montreal Neurological Institute (MNI) anatomical pipeline. Native MRI images were first registered into a standardized stereotaxic space using an affine transformation^36^. Non-uniformity artifacts were corrected using an N3 algorithm, and the registered and corrected volumes were classified as white matter, gray matter, cerebrospinal fluid, and background using an artificial neural net classifier^37, 38^. The surfaces of the inner and outer cortices were automatically extracted by deforming a spherical mesh onto the gray/white boundary in each hemisphere, using the Constrained Laplacian-Based Automated Segmentation with Proximities algorithm, which has been well-validated and extensively described elsewhere^39, 40^.

Cortical thickness was calculated as the Euclidean distance between the linked vertices of the inner and outer surfaces, after applying an inverse transformation matrix to cortical surfaces and reconstructing them in the native space^40, 41^. To control for brain size, we computed intracranial volume (ICV) using classified tissue information and a skull mask, which was acquired from a T1-weighted image^42^. ICV was defined as the total volume of gray matter (GM), white matter (WM), and cerebrospinal fluid (CSF) with consideration of voxel dimension. Classified GM, WM, CSF, and background within the mask were transformed back into the individual native space.

To compare the thicknesses of corresponding regions among the subjects, the thicknesses were spatially registered on an unbiased iterative group template by matching sulcal folding patterns using surface-based registration that performs sphere-to-sphere warping^43, 44^. For global and lobar regional analyses, we used the lobe-parcellated group template that had been divided into frontal, temporal, parietal, and occipital lobes using SUMA (http://afni.nimh.nih.gov), as in a previous study^45^. The averaged values for the thickness of the whole vertex in each hemisphere and lobar region were used for the global analysis.

### Assessment of cognitive function

All patients were annually followed up with Seoul neuropsychological screening battery. Language function was assessed by Korean version of the Boston Naming Test score. Visuospatial function was assessed by Rey-Osterrieth Complex Figure Test (RCFT) copy score. Memory function was assessed by summing scores of verbal memory (Seoul Verbal Learning Test immediate recall, delayed recall, recognition score) and visual memory tests (RCFT immediate recall, delayed recall, recognition score). Frontal-executive function was assessed by summing scores of category word generation test, phonemic word generation test, and the Stroop color reading test. General cognition was assessed by the Korean version of the Mini-Mental Status Examination (K-MMSE) and the Clinical Dementia Rating-Sum of Boxes (CDR-SOB) test.

### Statistical analysis

To evaluate whether APOE genotype affects longitudinal changes in PiB SUVR, lacune number, cortical thickness, or cognitive function, an interaction between participant group (APOE2 carriers, APOE3 homozygotes, or APOE4 carriers) and follow-up time (years) from baseline assessment (group-by-time) was examined with a generalized estimating equation, controlling for age and gender. We further controlled for intracranial volume (ICV) in the analysis of cortical thickness and controlled for education in the analysis of cognitive function. Since the baseline WMH volume was significantly lower in APOE4 carriers, we performed additional analysis after further controlling for the baseline WMH volume. A two-sided p-value < 0.05 was considered statistically significant. All analyses were performed using PASW Statistics 17 software (SPSS Inc., Chicago, IL, USA)

In order to compare cortical thickness topography, we used the Surfstat package created by Dr. Keith Worsley (http://www.math.mcgill.ca/keith/surfstat). Localized cortical thickness differences between APOE2 carriers and APOE3 homozygotes were analyzed using a general linear model (GLM) on a vertex-by-vertex basis. Group-by-time interactions were tested with the GLM mixed effect model for cortical thickness at every vertex accounting for age, gender and ICV effects. The cortical surface model contained 81,924 vertices. Statistical significance for an individual voxel was assumed at a p value less than 0.01 (uncorrected for multiple comparisons).

## Electronic supplementary material

Supplementary Information
