# Supplementary material for: Protective effects of APOE e2 against disease progression in subcortical vascular mild cognitive impairment patients: A three-year longitudinal study
Source: Sci Rep. 2017 May 15;7:1910. doi: 10.1038/s41598-017-02046-y (PMC5432504; doi:10.1038/s41598-017-02046-y)
Supplement: Supplementary file 1 — Supplementary Information [file 41598_2017_2046_MOESM1_ESM.doc]

**Protective effects of *APOE e2* against disease progression in subcortical vascular mild cognitive impairment patients: A three-year longitudinal study**

Yeo Jin Kim^a,b,c^, Sang Won Seo^b,c,l^ Seong Beom Park^b,c^, Jin Ju Yang^d^, Jin San Lee^e^, Juyoun Lee^b,c,f^, Young Kyoung Jang^b,c^, Sung Tae Kim^g^, Kyung-Han Lee^h^, Jong Min Lee^d^, Jae-Hong Lee^i^, Jae Seung Kim^j^, Duk L. Na^b,c,k^, Hee Jin Kim^b,c^

^a^ Department of Neurology, Chuncheon Sacred Heart Hospital, Hallym University College of Medicine, Chuncheon, Korea

^b^Department of Neurology and ^g^Radiology, Sungkyunkwan University School of Medicine, Samsung Medical Center, Seoul, Korea

^c^Neuroscience Center, Samsung Medical Center, Seoul, Korea,

^d^Department of Biomedical Engineering, Hanyang University, Seoul, Korea

^e^Department of Neurology, Kyung Hee University Hospital, Seoul, Korea

^f^Department of Neurology, Chungnam National University Hospital, Daejeon, Korea

^h^Department of Nuclear Medicine, Sungkyunkwan University School of Medicine, Samsung Medical Center, Seoul, Korea

^i^Department of Neurology and ^j^Nuclear Medicine, Asan Medical Center, University of Ulsan College of Medicine, Seoul, Korea

^k^Department of Health Sciences and Technology, and ^l^Department of Clinical Research Design & Evaluation, SAIHST, Sungkyunkwan University, Seoul, Korea

Corresponding author: Hee Jin Kim, MD, PhD (E-mail: [evekhj@gmail.com](mailto:evekhj@gmail.com))

**
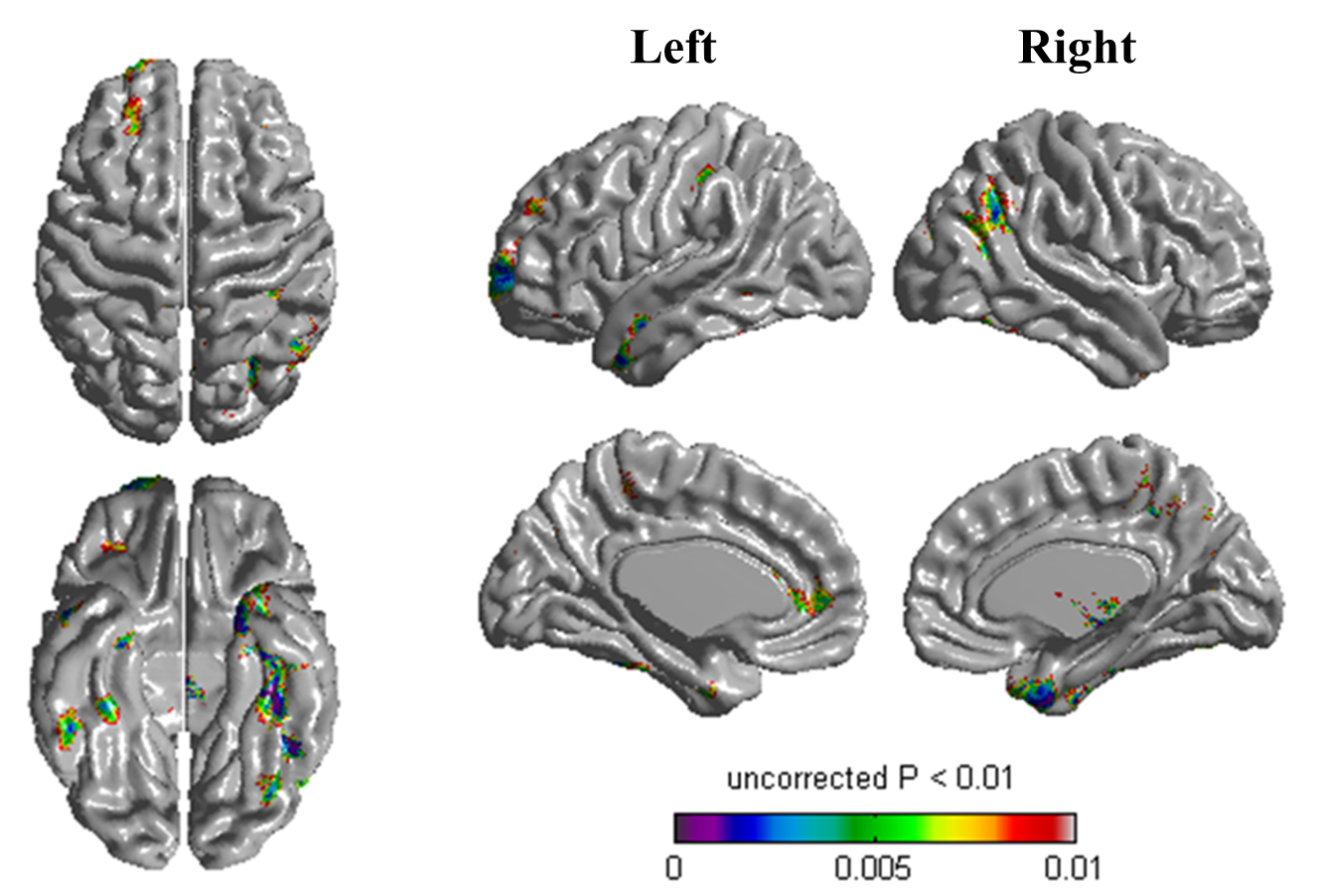
**

**Supplementary Figure S1.** **Statistical map shows regions where APOE2 carriers had slower rate of cortical thinning compared to APOE3 homozygotes.** Compared to the APOE3 homozygotes, APOE2 carriers showed slower cortical thinning in the left dorsolateral frontal, lateral temporal, medial frontal; right lateral parietal, medial temporal; and bilateral inferior temporal areas. Linear mixed effects models were performed using group (APOE genotype), time, age, gender, baseline WMH volume, intracranial volume, and the interaction term between group and time (group-by-time) as fixed effects; and patient as a random effect (uncorrected p< 0.01).

Abbreviation: APOE, apolipoprotein E; WMH, white matter hyperintensity


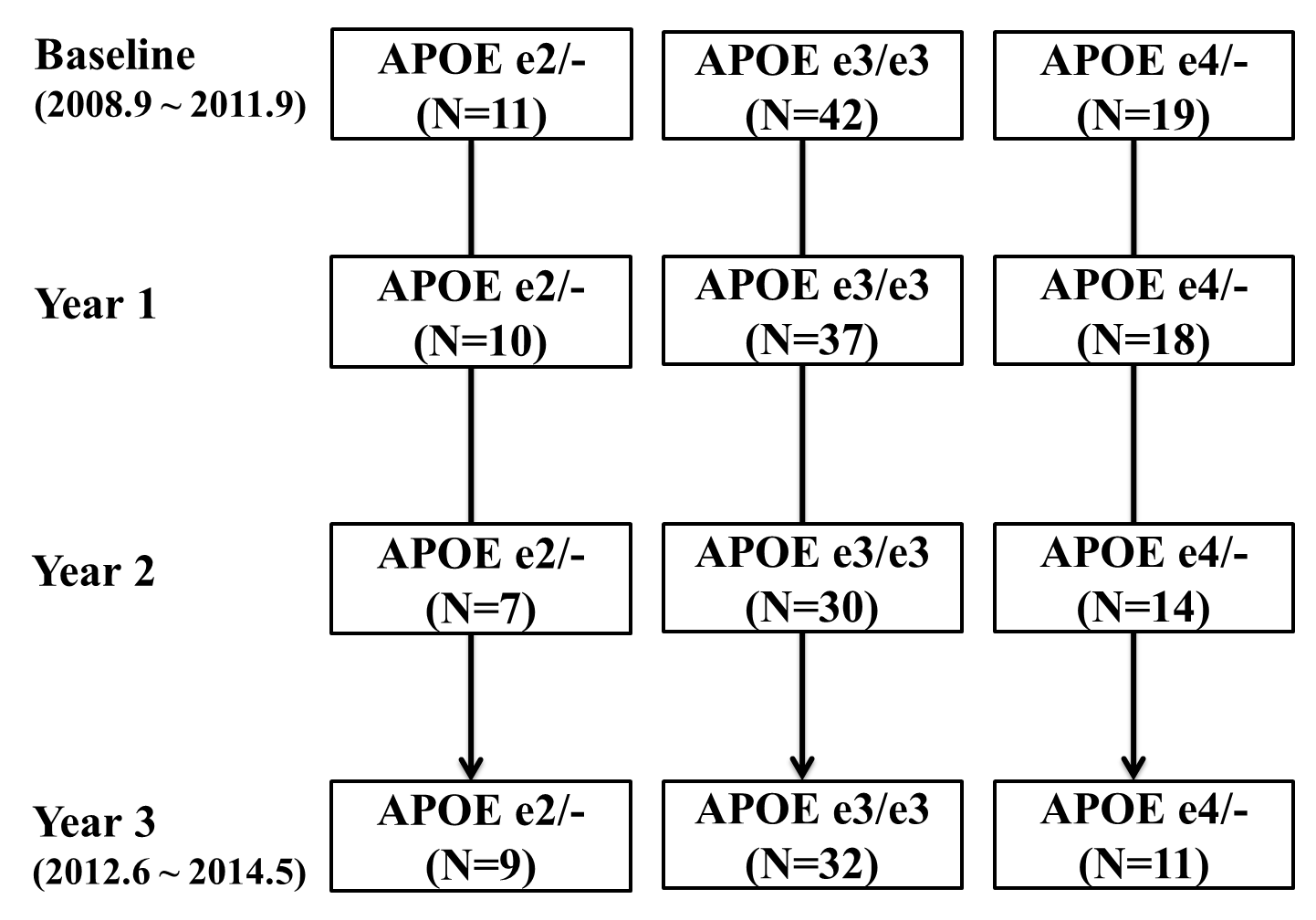


**Supplementary Figure S2.** **Flow chart showing the number of participants in each year**

Abbreviation: APOE e2/-, apolipoprotein E e2 carriers; APOE e3/e3, apolipoprotein E e3/e3 homozygotes; APOE e4/-, apolipoprotein E e4 carriers
